# Gender-based differences in the high-risk sexual behaviours of young people aged 15-29 in Melilla (Spain): a cross-sectional study

**DOI:** 10.1186/1471-2458-14-745

**Published:** 2014-07-23

**Authors:** Esperanza Romero-Estudillo, Emilio González-Jiménez, María C Mesa-Franco, Inmaculada García-García

**Affiliations:** Department of Nursing, Faculty of Nursing, University of Granada, C/ Santander N° 1, (52071) Melilla, Spain; Department of Evolutionary Psychology and Education, Faculty of Education Science, University of Granada, Campus Universitario de La Cartuja, s/n (18071), Granada, Spain; Department of Nursing, Faculty of Health Sciences, University of Granada, Av/ Madrid s/n (18071), Granada, Spain

**Keywords:** Gender, Sexual behaviour, Risk behaviour, Young people

## Abstract

**Background:**

Research confirms the existence of gender-based differences regarding the high-risk sexual behaviour (non-use of condoms and casual partners) of young men and women. The objectives were to provide evidence for this association; to analyse the reasons why both sexes have sexual relations with casual partners and to ascertain the motives for condom use or non-use during casual sex.

**Methods:**

A cross-sectional study was performed on a sample of 900 participants, 524 males and 376 females. All participants were 15-29 (20.93 ± 4.071) years of age and came from four different centres (a university, two secondary schools, and a military base) in Melilla (Spain). The participants were given a socio-demographic survey as well as a psychometric text on high-risk sexual behaviour.

**Results:**

The results found gender-based significant differences for *sexual relations with penetration* (*p* = 0.001), *number of sexual partners* (*p* = 0.001), and *sexual relations with casual partners* (*p* = 0.001). In all of these variables, male participants had higher percentages than female participants. Reasons for having casual sexual relations were also different for men and women, differences were found for the items, *opportunity* (*p* = 0.001), *interest in knowing the other person* (*p* = 0.015), *physical excitement* (*p* = 0.056) and *drug consumption* (*p* = 0.059). Regarding the reasons for consistent condom use with casual partners, there were differences for the item, *my demand of a condom* (*p* = 0.002). For the non-use of condoms with casual partners, differences were found for the items, *I do not like to use condoms* (*p* = 0.001) and *condoms lessen sensitivity and reduce pleasure* (*p* = 0.009).

**Conclusions:**

Men and women were found to have different high-risk sexual behaviours and practices. Of the motives for having sexual relations with casual partners, male participants considered *opportunity* and *interest in knowing the other person* to be more important than the female participants. Regarding condom use, the female participants’ demand to use a condom was a significant gender-based difference. In contrast to the young women, the male participants mostly justified not using a condom because it lessened sensitivity and reduced pleasure.

## Background

Research suggests that males and females express their sexuality differently [1] and behave differently when engaging in high-risk sexual practices. More specifically, young men tend to become sexually active earlier. They also have a higher number of casual partners than young women [2]. In contrast, females usually have sexual relations within the context of a stable committed relationship, which is associated with love and trust [3]. They seem to feel a greater need of a stable partner to have sexual relations and tend to place a greater value on his/her faithfulness [4]. Women also tend to value affection and social position, whereas for men physical attractiveness is important [4]. In the same line, Larrañaga et al. [5] found that Spanish university students have gender-based sexual attitudes that make them reluctant to use contraceptive methods, particularly condoms. An increase in macho as well as romantic attitudes in young people appears to contribute to the rejection of preventive measures in sexual relations.

According to Falah-Hassani et al. [6], young women tend to be better informed about the risks of oral contraceptives (e.g. venous thromboembolic diseases) than their male counterparts. Hooke et al. [7] found that 73% of young women took charge of the use of contraceptives in contrast to 46% of young men. In addition, males did not have a negative perception of casual sex. This type of sexual relation occurs between people who are strangers or who are only slightly acquainted with each other [8].

According to García-Carpintero et al. [9], barrier contraceptives (e.g. condoms) are more frequently used by males and adolescents, mainly at the beginning of sexual relations with a casual partner. Moreover, the fact that the condom is considered to be a male contraceptive signifies that man tend to use it more than women.

The study conducted by Rodríguez Carrión et al. [10] on sexual behaviours in a sample of 2,225 adolescents found that condoms were the most frequently used contraceptive followed by birth control pills. Of the sexually active participants, 2% used no contraceptive method, 4% used the withdrawal method, and 16.6% reported using the morning-after pill.

Hooke et al. [7] also identified gender-related differences regarding the avoidance of pregnancy to explain the fact that females assumed greater responsibility for birth control than males. Other factors that influenced high-risk sexual practices (non-use of condom and casual partners) included the existence of physical attraction or of an affective relationship. Also mentioned were the level of sexual excitement during relations and the consumption of stimulants such as alcohol or drugs [11, 12].

With regard to protection (STIs and pregnancy), Planes et al. [13] reported that the increased use of oral contraceptives has led to a corresponding decrease in the use of condoms. This suggests that young people, in particular, women, are more worried about avoiding pregnancy than about becoming infected with the human immunodeficiency virus (HIV) or contracting a sexually transmitted infection (STI). This indicates that young people believe that the main problem related to their sexuality is risk of pregnancy. Another explanation for the non-use of condoms could be the existence of a stable relationship.

One of the main reasons given by the participants for not using condoms included the lessening of sensitivity and sexual pleasure as well as the fear of being rejected by one’s partner because of a lack of trust [14]. This situation has contributed to a dramatic rise in the prevalence of STIs in the youth and adult population. Other practices that could explain this situation are oral and anal sex [15, 16].

In fact, it is estimated that there are 333 million new cases in the world each year [17–19]. In recent years, the prevalence of STIs in adolescents living in the city of Melilla, Spanish exclave on the coast of North Africa, has soared. According to data published in the first semester of 2012 by the National Institute of Statistics (Spain) [20], there are 90 diagnosed cases of HIV, due to injection drug use (62.2%), heterosexual contact (17.7%), and homosexual contact involving men having sex with men (MSM) (10.2%). Other transmission categories are blood transfusion (2.2%) and mother-to-child vertical transmission (2.2%). Of the patients diagnosed, 78.8% are males and 21.1% are females.

Furthermore, according to the Ministry of Health and Social Policy in Spain [21], in 2012 there were 109 voluntary interruptions of pregnancy (VIPs) in Melilla. Ten of these cases were females 15-19 years of age; 36 cases were females 20-24 years of age; 31 were females 25-29 years of age; and 32 were females 30-44 years of age. Most of the women were single, and 30.27% had had at least one previous VIP. Interestingly, in Ceuta, a Spanish city also located in North Africa, whose sociodemographic and cultural characteristics are very similar to those of Melilla, there were only 38 cases of VIPs during the same time period. This difference in the number of VIPs is one of the reasons why it was decided to investigate sexual behaviour in Melilla.

The steadily increasing prevalence of STIs in people of younger ages underlines the need to examine the aspects involved in the adoption of high-risk sexual practices, based on gender [22]. Accordingly, the first objective of this study was to identify a possible association between gender and high-risk sexual practices. The second objective was to analyse the motives of both males and females for having sexual relations with casual partners. The third was to determine the aspects that conditioned condom use or non-use in casual sex.

## Methods

### Study design and sampling

A cross-sectional study was carried out in Melilla at two secondary schools, a branch of the National Distance Education University (UNED), the campus of the University of Granada, and a Spanish military base. In order to calculate optimal sample size, we consulted the ongoing census of the National Institute of Statistics (Spain), for 1 January 2013 [23]. According to the census data, Melilla has 17,998 residents that are 15-29 years of age [24]. Assuming an error of 3%, it was estimated that our study required a sample population of approximately 1000 participants of 15-29 years of age. The sample was finally composed of 900 subjects: 524 males (58.2%) and 376 females (41.8%).

All participants were 15-29 (20.93 ± 4.071) years of age and had previously agreed to answer 80% of the questions in the survey. Participants were excluded if they did not fall into this age range or if they refused to respond to the required number of items.

The study was carried out in three phases. During the first phase (6.5 months), the researchers obtained permission to access the secondary schools, universities, and military base where the data were to be collected. Meetings were held with the directors of each of the centres to explain the objectives of the study and to schedule a date for a meeting with parents or guardians of students under 18. During the second phase of the project (four months), meetings were held with parents or guardians. After an explanation of the study, they were given an informed consent form. During the third phase (six months), the participants were asked to fill out two questionnaires.

Two data collection instruments were used. The first questionnaire collected socio-demographic data (age, gender, marital status, etc.), and the second specifically evaluated psychological variables and the high-risk sexual behaviour of the participants. This instrument, created by Piña et al. [25], is based on Ribes’s Psychological Model of Biological Health [26] for the prevention of illnesses. It includes historical and contextual variables (motives, biological states, and social situations) that describe and explain high-risk sexual behaviours. Variables with an impact on sexual behaviour included motivations, substance consumption (alcohol and drugs), and the influence of other individuals. The questionnaire contained 11 demographic items and 28 risk-behaviour items to which the participant could only give one response in the different categories for each item. In the case of the variable *sexual relations with penetration*, the item did not specify the type of penetration.

There were also 76 questions on contextual variables (i.e. motives, social situations, and biological states) where the participant had the choice of three or four responses. For example, for *physical attraction* as a motive for having sexual relations with casual partners, participants could enter one of the following response options: *Highly determining, Fairly determining, Slightly determining and Not determining*. Here, *determining* was defined as something (in this case, sexual attraction) that contributes to producing a result or behaviour (sexual relations). The modifiers *highly, fairly, slightly,* and *not determining* designate a four-point scale.

The conceptual validity of this instrument [25] was obtained by means of exploratory factor analysis with varimax rotation. There were three factors (i.e. readiness for sexual relations, motives for the non-use of condoms, and motives for condom use) with individual values higher than 1, which altogether accounted for 38.36% of the total variance, In the reliability analysis, the value of Cronbach’s alpha was 0.82

### Statistical analyses

A descriptive analysis was performed by using percentages and frequencies for the category variables, mean, and typical deviation in the quantitative variables and contingency tables. A bivariate analysis of these data was performed, using chi-square and Mann-Whitney U tests. *P* < 0.05 was considered significant.

### Ethics

This research was performed in strict compliance with the international code of medical ethics established by the World Medical Association and the Declaration of Helsinki. Ethical approval for the conduct of the study was obtained from the University of Granada. Data gathering processes followed standard ethical guidelines. Participants were assured that participation was voluntary and that the information provided by them would be kept completely anonymous and confidential. The participants (15-17 years of age) had the written authorization of their parents or guardians to participate in the study whereas subjects that were 18 and older personally gave their written informed consent.

## Results

The sample population had a mean age of 20.93 years (SD 4.71), and was distributed in the following intervals: 364 (40.4%) of the participants were 15-19 years old; 345 (38.3%) were 20-24 years old; and 191 (21.2%) were 25-29 years old. Of these participants, 664 (73.9%) were students; 137 (15.2%) were in the armed forces; 33 (3.7%) had jobs; and 65 (7.2%) were unemployed.

Regarding sexual practices, according to gender (Table 1), the results showed that 59% of the participants (of a sample composed of 93.5% heterosexuals, 3.5% homosexuals, 3% bisexuals) had had sexual intercourse with penetration. This was true for 78.7% of the male participants, which meant that there were statistically significant differences (*p* = 0.001) between the two sexes. In reference to the variable, *condom use during the first sexual relation,* there were no significant *gender* differences though a slightly higher percentage of men (79.1%) than women (78%) reported condom use in the first relation.

**Table 1:** **High-risk sexual practices, according to gender**

	Total	Females n (%)	Males n (%)	P value ^a^
**Sexual relations with penetration**				**0.001**
Yes	605 (67.2)	309 (59)	296 (78.7)	
No	295 (32.8)	215 (41)	80 (21.3)	
**Condom use during the first sexual relation**				**0.767**
Yes	475 (78.5)	241 (78)	234 (79.1)	
No	130 (21.5)	62 (22)	62 (20.9)	
**Frequency of condom use:**				**0.475**
Always	179 (29.8)	84 (27.5)	95 (32.3)	
Most of the time	231 (38.5)	119 (33.9)	112 (38.1)	
Rarely	153 (25.5)	81 (26.5)	72 (24.5)	
Never	37 (6.2)	22 (7.2)	15 (5.1)	
**Number of sexual partners:**				**0.001**
Only 1	171 (28.5)	116 (37.9)	55 (18.6)	
2-4	200 (33.3)	106 (34.6)	94 (31.9)	
5-7	93 (15.5)	42 (13.7)	51 (17.3)	
8 or more	137 (22.8)	42 (13.7)	95 (32.2)	
**Sexual relations with casual partners:**				**0.001**
Yes	290 (66.1)	107 (55.2)	183 (74.7)	
No	149 (33.9)	87 (44.8)	62 (25.3)	
**Frequency of condom use with a casual partner:**				**0.212**
Always	157 (53.8)	54 (49.1)	103 (56.6)	
Most of the time	74 (25.3)	26 (23.6)	48 (26.4)	
Rarely	46 (15.8)	22 (20.0)	24 (13.2)	
Never	15 (5.1)	8 (7.3)	7 (3.8)	

^a^:using chi-square test.

With regard to *frequency of condom use*, no significant differences were found (*p* = 0.475) between sexes. More specifically, 33.9% of the female participants and 38.1% of the male participants said that they used a condom most of the time. In reference to *number of sexual partners,* there were significant differences (*p* = 0.001) between both sexes. A total of 37.9% of the female participants reported intercourse with only one partner, in contrast to 32.2% of the male participants who said that they had had eight or more partners.

Significant gender differences were also found for *sexual relations with casual partners* (*p* = 0.001). In this regard, 55.2% of the women said that they had engaged in casual sex, in contrast to 74.7% of the men. For *frequency of condom use with casual partners,* there were no significant gender-based differences (*p* = 0.212). More specifically, 49.1% of the female participants and 56.6% of the male participants said that they always used a condom with casual partners.

With regards to the reasons for having casual sexual relations (Table 2), there were significant gender differences (*p* = 0.001) for the item *opportunity. Opportunity* in this context is understood as a time or condition favourable for a particular action (e.g. having casual sexual relations). It was found that 38.2% of the female participants regarded this item as fairly determining, whereas 63.7% of the males considered it to be very important. Consequently, for both males and females, *opportunity* was an important reason for engaging in casual sex. Significant differences between men and women were also found for the variable *interest in knowing the other person* (*p* = 0.015). For 30% of the women, this variable was slightly determining, whereas for 33.1% of the men, it was fairly determining.

**Table 2:** **Motives for men and women to have sexual relations with casual partners**

	Total	Females	Males	P value ^a^
**Opportunity:**				**0.001**
Highly determining	144 (49.3)	28 (25.5)	116 (63.7)	
Fairly determining	95 (32.5)	42 (38.2)	53 (29.1)	
Slightly determining	31 (10.6)	25 (22.7)	6 (3.3)	
Not determining	22 (7.5)	15 (13.6)	7 (3.8)	
**Interest in knowing the other person:**				**0.015**
Highly determining	61 (21)	17 (15.5)	44 (24.3)	
Fairly determining	91 (31.3)	31 (28.2)	60 (33.1)	
Slightly determining	77 (26.5)	33 (30)	44 (24.3)	
Not determining	62 (21.3)	29 (26.4)	33 (18.2)	
**Physical attraction:**				**0.835**
Highly determining	194 (66.9)	71 (64.5)	123 (68.3)	
Fairly determining	78 (26.9)	64.5 (31.8)	43 (23.9)	
Slightly determining	13 (4.5)	3 (2.7)	10 (5.6)	
Not determining	5 (1.7)	1 (0.9)	4 (2.2)	
**Physical excitement:**				**0.056**
Highly determining	167 (57.2)	54 (49.1)	113 (62.1)	
Fairly determining	97 (33.2)	43 (39.1)	54 (29.7)	
Slightly determining	13 (4.5)	6 (5.5)	7 (3.8)	
Not determining	15 (5.1)	7 (6.4)	8 (4.4)	
**Alcohol consumption:**				**0.227**
Highly determining	38 (13.1)	9 (8.2)	29 (16)	
Fairly determining	69 (23.7)	25 (22.7)	44 (24.3)	
Slightly determining	64 (22)	32 (29.1)	32 (17.7)	
Not determining	120 (41.2)	44 (40)	76 (42)	
**Drug consumption:**				**0.059**
Highly determining	20 (6.9)	4 (3.6)	16 (8.8)	
Fairly determining	15 (5.2)	5 (4.5)	10 (5.5)	
Slightly determining	23 (7.9)	8 (7.3)	15 (8.3)	
Not determining	233 (80.1)	93 (84.5)	140 (77.3)	

^a^:using the Mann-Whitney U Test.

In relation to *physical attraction,* there were no significant differences between men and women (*p* = 0.835) since for both, this factor was highly determining. However, in the case of *physical excitement,* there were significant gender-based differences (*p* = 0.056). In fact, this item was highly determining for 49.1% of the female participants. In other words, females regarded physical excitement as an important reason for having sexual relations with casual partners. This was also the case for 62.1% of the male participants.

*Alcohol consumption* was generally not regarded as influential, as reflected in the lack of significant differences (*p* = 0.227) for both sexes. In reference to this item, 22.7% of the women and 24.3% of the men said that it was fairly determining. However, there were significant differences with regards to *drug consumption,* despite the fact that 84.5% of the female participants and 77.3% of the male participants stated that this item was not determining.

In relation to the motives that led to the consistent use of condoms in casual sexual relations (Table 3), 84.5% of the women and 85.2% of the men affirmed that *avoidance of pregnancy* was very important. Thus, there were no significant gender differences (p = 0.954). Neither were there differences between sexes with regards to *prevention of STIs*. This item was also very important for 89.5% of the women and 86.2% of the men.

**Table 3:** **Motives for men and women to consistently use condoms in sexual relations with casual partners**

	Total	Females	Males	P value ^a^
**Avoidance of pregnancy**				**0.954**
Highly determining	141 (84.9)	49 (84.5)	92 (85.2)	
Fairly determining	15 (9)	6 (10.3)	9 (8.3)	
Slightly determining	4 (2.4)	2 (3.4)	2 (1.9)	
Not determining	6 (3.6)	1 (1.7)	5 (4.6)	
**Prevention of STIs**				**0.565**
Highly determining	145 (87.3)	51 (89.5)	94 (86.2)	
Fairly determining	15 (9)	4 (7)	11 (10.1)	
Slightly determining	3 (1.8)	1 (1.8)	2 (1.8)	
Not determining	3 (1.8)	1 (1.8)	2 (1.8)	
**My demand of a condom**				**0.002**
Highly determining	83 (50.6)	37 (64.9)	46 (43)	
Fairly determining	37 (22.6)	13 (22.8)	24 (22.4)	
Slightly determining	16 (9.8)	3 (5.3)	13 (12.1)	
Not determining	28 ( 17.1)	4 (7.0)	24 (22.4)	
**Partner demand of a condom**				**0.107**
Highly determining	38 (23.3)	11 (19.3)	27 (25.5)	
Fairly determining	51 (31.3)	14 (24.6)	37 (34.9)	
Slightly determining	37 (22.7)	17 (29.8)	20 (18.9)	
Not determining	37 (22.7)	15 (26.3)	22 (20.8)	

^a^:using the Mann-Whitney U Test.

However, for the variable, *my demand of a condom*, there were gender-based differences (p = 0.002). A total of 64.9% of the female participants regarded this item as highly determining in contrast to 43% of the male subjects. In the case when the partner requested the use of a condom (*partner’s demand of a condom*)*,* 29.8% of the women said that the fact that the other person asked to use a condom was slightly determining followed by 34.9% of the men who regarded it as fairly determining with regard to the consistent use of condoms with casual partners.

In reference to the motives for consistently not using a condom in sexual relations with casual partners (Table 4), there were significant differences between sexes (p = 0.001). For 50% of the women, the reason, *I do not like to use a condom* did not influence condom use. However, for 28% of the men, this reason was very important. Regarding the reason, *my partner refused to use one,* 44.8% of the women stated that this factor was not determining, followed by 30.5% of the men who regarded it as fairly determining or not determining.

**Table 4:** **Motives for men and women to consistently not use a condom with casual partners**

	Total	Females	Males	P value ^a^
**I do not like to use a condom**				**0.001**
Highly determining	31 (22.1)	8 (12.8)	23 (28)	
Fairly determining	31 (22.1)	9 (15.5)	22 (26.8)	
Slightly determining	30 (21.4)	12 (20.7)	18 (22)	
Not determining	48 (34.3)	29 (50)	19 (23.2)	
**My partners do not like to use a condom**				**0.409**
Highly determining	19 (13.6)	10 (17.2)	9 (11)	
Fairly determining	37 (26.4)	12 (20.7)	25 (30.5)	
Slightly determining	33 (23.6)	10 (17.2)	23 (28)	
Not determining	51 (36.4)	26 (44.8)	25 (30.5)	
**A condom lessens sensitivity and reduces pleasure**				**0.009**
Highly determining	61 (44.2)	19 (32.8)	42 (52.5)	
Fairly determining	30 (21.7)	12 (20.7)	18 (22.5)	
Slightly determining	22 (15.9)	13 (22.4)	9 (11.2)	
Not determining	25 (18.1)	14 (24.1)	11 (13.8)	
**I did not have a condom at the time**				**0.235**
Highly determining	47 (33.8)	23 (39.7)	24 (29.6)	
Fairly determining	35 (25.2)	14 (24.1)	21 (25.9)	
Slightly determining	19 (13.7)	7 (12.1)	12 (14.8)	
Not determining	38 (27.3)	14 (24.1)	24 (29.6)	

^a^:using the Mann-Whitney U Test.

However, there were significant differences between sexes for the reason, *a condom lessens sensitivity and reduces pleasure* (*p* = 0.009). Thus, for 32.8% of the female participants and 52.5% of the male subjects, this was a very important motive for not using condoms. For the reason, *I did not have a condom at the time,* there were no significant gender-based differences (*p* = 0.235). A total of 39.7% of the women and 29.6% of the men regarded this reason as very important in the consistent use of condoms with casual partners.

## Discussion

The results obtained in this study were similar to the findings of previous research [27, 28]. They identified the existence of an association between gender and high-risk sexual behaviour and practices. A high percentage of male heterosexual, homosexual, and bisexual participants reported sexual intercourse with penetration, and reported a greater number of sexual partners than the female participants. This is consistent with Bermúdez, et al. [29] and López, et al. [30], who highlight that male adolescents are more interested than female adolescents in sexual practices in affective relations. However, in this study, no significant differences were found in the use of condoms in the first sexual relation. This contrasts with previous research [31]. However, the number of men who declared that they had had sex with casual partners was considerably higher. This is consistent with other studies that found that men have a greater tendency to engage in high-risk sexual behaviour [32–34].

With regards to the participants’ reasons for having casual sex, our results found that there were significant differences that were gender-based. More specifically, *opportunity, interest in knowing the other person, physical excitement,* and *drug consumption* were found to be the main factors in which men and women differed with regard to having sexual relations with casual partners.

In a study of a sample of 423 university students, Piña & Rivera [35] found that young men who practiced sexual relations with strangers had a social motivation (disinhibition and alcohol or drug consumption). In contrast to the young women, the motivation was biological since they engaged in casual sex because they felt physically excited. These results are consistent with Valdés et al. [36], who found that this high-risk sexual practice is increasingly prevalent among adolescents. In many cases, it is due to a lack of information about the health risks involved. Moreover, in a study of 1559 university students, Robles et al. [37] found the subjects who engaged in casual sex more frequently adduced reasons for not using protection.

The results of our study showed that alcohol consumption was not a determining factor for having sexual relations with casual partners. In contrast, research carried out in other countries (United States, Colombia, and the United Kingdom) [38, 39] reported a correlation between alcohol consumption and an increase in high-risk sexual practices in young people. Similarly, studies have also found that when there is the prospect of a sexual encounter, young people may consume drugs in larger quantities and thus could be more disposed to high-risk sexual behaviours [40, 41]. Consequently, young people may not be aware of the risk involved when they engage in sexual relations under the influence of alcohol or any other drug.

Regarding the use of condoms with casual partners, our results showed significant differences between men and women. The most important reason for using a condom during casual sex was that the partner requested its use during the sexual relation (*partner’s demand of a condom*). These results support those of previous research [42, 43]. Similarly, in a study of a sample of 3530 university students, Ballester et al. [44] concluded that the young women perceived themselves as having a higher level of self-efficacy. In this context, this means that they had greater belief in their decision-making capacity with regards to condom use. In contrast, men only felt more competent when it was a question of purchasing condoms.

The main factors that motivated the non-use of condoms in casual sex were *I do not like to use a condom* and *a condom lessens sensitivity and reduces pleasure*. In fact, this last reason was considered to be very important for 32.8% of the women and 52.5% of the men. These results support the data collected in other studies [45]. The non-use of condoms may or may not vary, according to gender as found in previous research [46, 47].

Similarly, Dávila et al. [48] state that the motives for condom use can interfere with preventive behaviours. Fierros et al. [49] studied sexual activity with casual partners in a sample population of university students. They found that the female participants continued to practice high-risk sexual behaviours that could lead to an HIV infection since there was little possibility of their adopting protective measures with casual partners with whom they were barely acquainted. Feelings of attraction and momentary physical excitement prevented the women from engaging in protected sex.

### Limitations of the study

The main limitations of this study were its cross-sectional nature combined with the lack of data with regard to the training received by the participants and their sexual knowledge. Other important limitations were the small size of the sample and the collection of information by self-reporting, especially in reference to such a delicate topic. In future research it will be necessary to take into account the multicultural identity of the territory since this could determine conclusions in relation to sexual tendencies.

## Conclusions

Men and women engage in high-risk sexual practices and behaviours for very diverse reasons. It is necessary to consider these reasons in the design and implementation of preventive measures. Concerning the motives for having sexual relations with casual partners, male participants considered *opportunity* and *interest in knowing the other person* to be more important than the female participants. Regarding condom use, the female subjects’ demand of condom use was a significant gender-based difference. In contrast to the young women, the male participants mostly justified not using a condom because it lessened sensitivity and reduced pleasure. There are similarities between the results obtained in this study and those of other studies of sexual partners and gender. However, concerning motives for engaging in sexual relations, the results of previous research are far from uniform. In this sense, health promotion programs in education centers should recommend and sponsor activities that make young people aware of the need to use condoms in sexual contacts. In this sense, it is necessary to continue working to eradicate high-risk sexual behaviour and to foment a more equal, satisfying, and safer sexuality. Future research will evaluate the impact of educational programs on young people and their sexual behaviour.

## Abbreviations

HIV: Human immunodeficiency virus
STI: Sexually transmitted infections
UNED: National Distance Education University
INE: National Institute of Statistics.
